# Unveiling the nexus of postoperative fever and delirium in cardiac surgery: identifying predictors for enhanced patient care

**DOI:** 10.3389/fcvm.2023.1237055

**Published:** 2023-11-10

**Authors:** Ya-peng Wang, Bei-bei Shen, Cui-cui Zhu, Li Li, Shan Lu, Dong-jin Wang, Hua Jin, Qi Liu, Zhe-yun Wang, Min Ge

**Affiliations:** ^1^ Department of Cardio-Thoracic Surgery, Nanjing Drum Tower Hospital, Chinese Academy of Medical Sciences & Peking Union Medical College, Nanjing, China; ^2^ Department of Cardio-Thoracic Surgery, Nanjing Drum Tower Hospital, The Afliated Hospital of Nanjing University Medical School, Nanjing, China; ^3^ Department of Critical Care Medicine, Xiangya Hospital of Central South University, Changsha, China

**Keywords:** cardiac surgery, fever, CPB, postoperative delirium, prediction model

## Abstract

**Background:**

Postoperative delirium (POD) is a significant complication observed in cardiac surgery patients, characterized by acute cognitive decline, fluctuating mental status, consciousness impairment, and confusion. Despite its impact, POD often goes undiagnosed. Postoperative fever, a common occurrence after cardiac surgery, has not been comprehensively studied in relation to delirium. This study aims to identify perioperative period factors associated with POD in patients undergoing cardiopulmonary bypass, with the potential for implementing preventive interventions.

**Methods:**

In a prospective observational study conducted between February 2023 and April 2023 at the Department of Cardio-Thoracic Surgery, Nanjing Drum Tower Hospital, Affiliated Hospital of Nanjing University Medical School, a total of 232 patients who underwent cardiac surgery were enrolled. POD assessment utilized the Confusion Assessment Method for the ICU (CAM-ICU), while high fever was defined as a bladder temperature exceeding 39°C. Statistical analysis included univariate and multivariate analyses, logistic regression, nomogram development, and internal validation.

**Result:**

The overall incidence of postoperative delirium was found to be 12.1%. Multivariate analysis revealed that postoperative lactate levels [odds ratio (OR) = 1.787], maximum temperature (OR = 11.290), and cardiopulmonary bypass time (OR = 1.015) were independent predictors of POD. A predictive nomogram for POD was developed based on these three factors, demonstrating good discrimination and calibration. The prediction model exhibited a C-statistic value of 0.852 (95% CI, 0.763–0.941), demonstrating excellent discriminatory power. Sensitivity and specificity, based on the area under the receiver operating characteristic (AUROC) curve, were 91.2% and 67.9%, respectively.

**Conclusion:**

This study underscores the high prevalence of POD in cardiac surgery patients and identifies postoperative lactate levels, cardiopulmonary bypass duration, and postoperative fever as independent predictors of delirium. The association between postoperative fever and POD warrants further investigation. These findings have implications for implementing preventive strategies in high-risk patients, aiming to mitigate postoperative complications and improve patient outcomes.

## 1. Introduction

Delirium is a disease characterized by acute cognitive decline, fluctuating mental status, consciousness impairment, lack of attention, or confusion (1). It is a recognized adverse prognostic marker in intensive care unit (ICU) patients, associated with increased incidence, mortality, and the development of long-term neurocognitive deficits (2). The incidence of postoperative delirium in cardiac surgery patients ranges from 16% to 73% (3–7), and is associated with early postoperative mortality, prolonged hospitalization, discharge to long-term care facilities, functional and cognitive decline, and increased healthcare costs (8, 9). While delirium is typically considered a short-term cognitive impairment, long-term consequences, such as functional and cognitive decline, are possible.

Given the high incidence of postoperative delirium in cardiac surgery patients, and its impact on functional outcomes and quality of life, it is essential to study the association between delirium and functional outcomes in this population. However, cardiac surgeons, anesthesiologists, intensivists, and nurses may fail to recognize delirium in up to 84% of patients (10, 11). Although the EuroSCORE, a cardiac surgical risk assessment system, is associated with postoperative mortality and delirium, no studies have reported on the association between postoperative fever and delirium (7, 12, 13). Postoperative fever is more common in cardiac surgery and is associated with increased cerebral embolic load after cardiopulmonary bypass and increased release of chemotactic factors (14).

Therefore, this study aims to determine the preoperative, operative, and postoperative fever-related factors associated with postoperative delirium in patients undergoing cardiopulmonary bypass. Successful identification of these factors could lead to preventative interventions in high-risk patients, with the hope of preventing subsequent complications.

## 2. Materials and methods

### 2.1. Study design

Participants between February 2023 and April 2023, we conducted a prospective observational study at the Department of Cardio-Thoracic Surgery. The study was ethically approved by the institutional review board was registered on the Chinese Clinical Trial Registry (ChiCTR2000038762).

### 2.2. Participants

**Figure 1:**
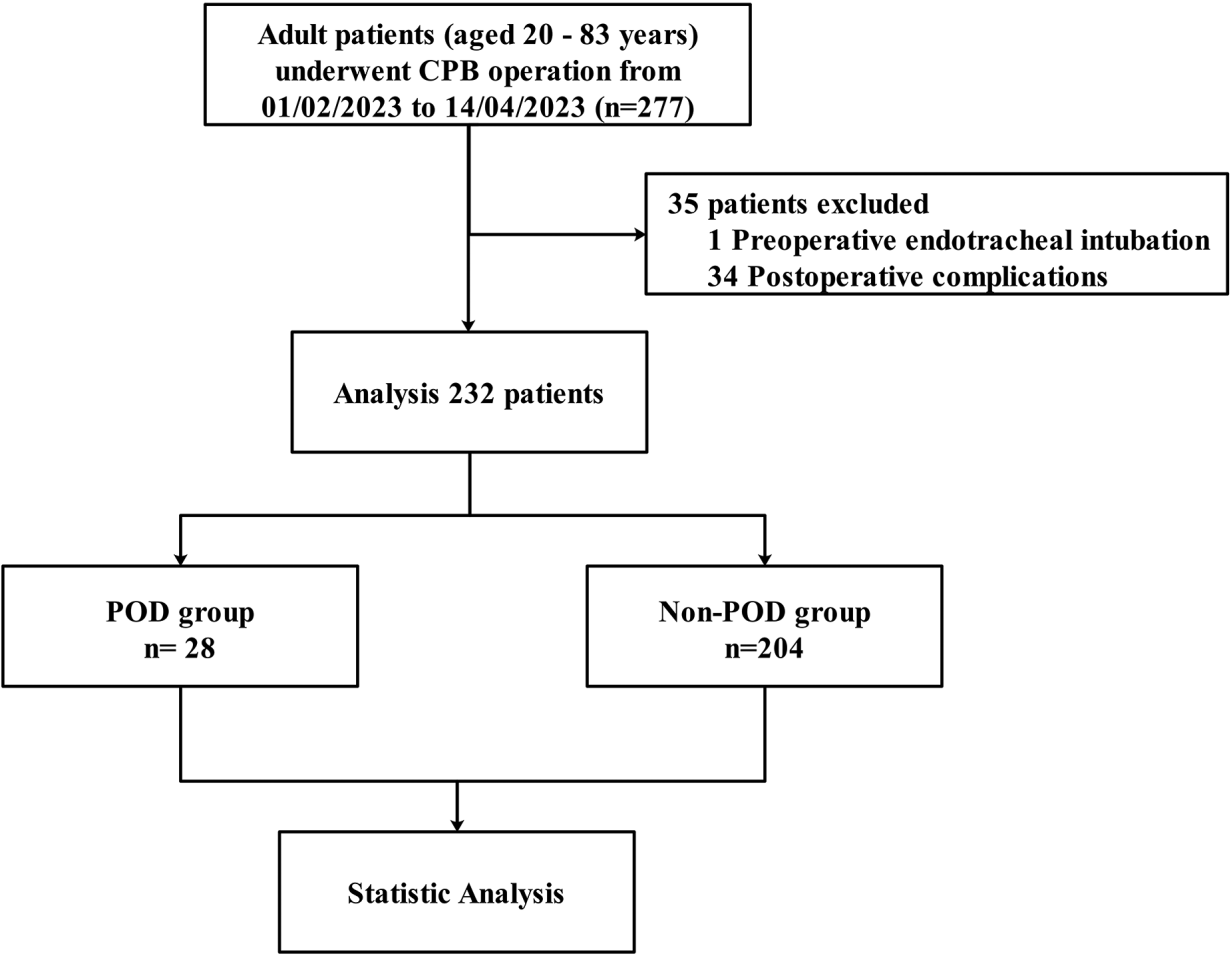
Study flowchart.

Between February 1, 2023, and April 14, 2023, this study recruited patients who underwent cardiac surgery under general anesthesia for cardiopulmonary bypass with the inclusion criteria of being admitted to the Cardio-Thoracic Surgery ICU for more than 24 h and providing written informed consent after receiving information sheets and potential risk disclosures. Exclusion criteria included patients under the age of 18, those diagnosed with delirium and stroke during admission, those with a Glasgow Coma Scale score of ≤8 points who required intubation and mechanical ventilation, those who were deeply sedated (as determined by Richmond Agitation Sedation Scale scores of −4 and −5), and those with alcohol withdrawal reactions (15), patients with infective endocarditis and preoperative fever. A total of 232 eligible patients were enrolled (Figure 1), and measures were taken to ensure the validity and robustness of the research findings.

Delirium is a challenging condition to diagnose in the intensive care unit (ICU), and many patients may go unrecognized. Despite the use of many delirium assessment instruments in published studies, the most widely used instrument is the Confusion Assessment Method (CAM) (16). CAM has a sensitivity of 94% and specificity of 89% compared to the gold standard diagnosis by psychiatrists (17). CAM-ICU was developed to accurately diagnose delirium in ICU patients who are often unable to speak due to mechanical ventilation. CAM-ICU has a sensitivity of 95% and specificity of 89% (16, 18).

### 2.3. Definition of end-points

In surgical patients who do not have permanent neurological impairment, postoperative delirium (POD) is identified using the Confusion Assessment Method for the ICU (CAM-ICU) (18) and evaluated twice daily for seven consecutive days. This method allows for consistent monitoring and diagnosis of POD, which is a common complication after surgery and can have significant negative effects on patient outcomes (19).

We define high fever as bladder temperature greater than 39°C and continuously monitor body temperature for 24 h postoperatively. This definition of high fever allows for consistent and accurate measurement of postoperative fever, which can indicate a pathological state and may require further evaluation and treatment.

### 2.4. Statistical analysis

In this study, we conducted a comprehensive analysis of the data using a range of statistical methods. Continuous variables were assessed for normality using the Kolmogorov–Smirnov test and reported either as mean ± standard deviation or median with interquartile ranges (Q1–Q3), depending on their distribution. Student's *t*-test and Mann–Whitney *U*-test were used to analyze normally and non-normally distributed continuous variables, respectively. Categorical variables were presented as frequencies and percentages and analyzed using either chi-squared test or Fisher's exact test. All statistical analyses were two-tailed, and a *P*-value less than 0.05 was considered statistically significant. Additionally, we employed single-variable binary logistic regression analysis to assess the relationship between various variables and the outcome, calculating odds ratios and 95% confidence intervals.

### 2.5. Model development

After conducting univariate analysis, variables that demonstrated statistical significance with a *P*-value less than 0.05 were included in a stepwise (backward: conditional) multivariate logistic regression analysis model to establish a predictive model. Furthermore, we constructed a nomogram using the variables with a *P*-value less than 0.05 in the multivariate analysis to facilitate clinical decision-making. This approach allowed us to identify the most significant predictors of the outcome of interest and develop a useful tool to aid in clinical management.

### 2.6. Model performance and internal validation

To assess the performance of the developed nomogram for predicting the probability of postoperative neurological complications in aortic surgery, we conducted internal validation using the bootstrap method with 1,000 resamples, evaluating both discrimination and calibration. The discrimination ability was assessed using the C-statistic, equivalent to the area under the receiver operating characteristic (ROC) curve (20). Calibration was assessed by plotting calibration curves and calculating the Brier score, which is the squared difference between observed and predicted probabilities (21). Furthermore, we conducted a decision curve analysis (DCA) to assess the clinical usefulness of the nomogram across various threshold probabilities. The statistical analysis was performed using IBM SPSS Statistics 26 and R.4.2.2, and significance was determined at *P* < 0.05. This rigorous approach enabled us to gain valuable insights into the data and identify potential predictors of the outcome of interest.

## 3. Results

### 3.1. Patients baseline characteristics

During the research period from February 1, 2023, to April 14, 2023, our analysis included a total of 232 patients, as shown in Figure 1. Preoperative risk factors, such as age, gender, BMI, hypertension, NYHA class, as well as important intraoperative risk factors, including surgical category and cardiopulmonary bypass time (CPB), were identified, as presented in Table 1. The overall incidence of POD was found to be 12.1%, as shown in Table 1, and was significantly associated with CPB and postoperative lactate levels. Moreover, there were significant variations in Tmax between the POD and Non-POD groups. POD was also found to be related to ICU length of stay.

**Table 1:** Basic characteristics in POD and Non-POD groups.

Characteristic	POD (*N* = 28)	Non-POD (*N* = 204)	*P*-value
Sex male (*N*, %)	15 (53.6)	114 (55.9)	0.817
Age (years)^a^	60.50 (50.0–64.0)	61.00 (52.0–69.0)	0.294
BMI (kg/m^2^)^a^	25.0 (22.6–26.5)	23.43 (21.46–26.89)	0.421
Hypertension (*N*, %)	16 (57.1)	114 (55.9)	0.900
CAD (*N*, %)	6 (21.4)	63 (30.9)	0.305
Hepatitis (*N*, %)	2 (7.1)	8 (3.9)	0.771
Renal insufficiency (*N*, %)	1 (3.6)	7 (3.4)	1.000
Atrial fibrillation (*N*, %)	11 (39.3)	59 (28.9)	0.263
NYHK			0.301
Ⅰ (*N*, %)	3 (11.1)	22 (10.8)	
Ⅱ (*N*, %)	9 (33.3)	48 (23.6)	
Ⅲ (*N*, %)	15 (55.6)	122 (60.1)	
Ⅳ (*N*, %)	0 (0.0)	11 (5.4)	
Smoking (*N*, %)	4 (14.3)	38 (18.6)	0.576
Alcohol (*N*, %)	2 (7.1)	28 (13.7)	0.501
Surgery procedure			0.473
Valve surgery (*N*, %)	9 (32.1)	87 (42.6)	
CABG (*N*, %)	0 (0.0)	9 (4.4)	
Valve + CABG	2 (7.1)	17 (8.3)	
Valve + Maze operation	8 (28.6)	38 (18.6)	
Aortic surgery	7 (25.0)	43 (21.1)	
Congenital heart disease	2 (7.1)	10 (4.9)	
CBP time (min)^a^	168.00 (135.5–193.5)	122.00 (96.0–158.0)	0.0001
Lactate^a^	2.35 (1.53–3.60)	1.5 (1.20–2.10)	0.0001
Tmax (*N*, %)	20 (71.4)	34 (16.7)	0.0001
CCU day^a^	6.00 (4.00–8.00)	3.00 (2.00–4.00)	<0.0001
Length of stay^a^	19.50 (16.25–26.00)	18.00 (15.25–22.00)	0.151

BMI: body mass index; CAD: coronary artery disease; CABG: coronary artery bypass grafting; CPB: cardiopulmonary bypass.

^a^ Values are expressed as interquartile spacing [median (¼–¾ digits)].

### 3.2. Identifying predictors

The results of multivariate analysis for POD are listed in Table 2, For this phase of the analysis, three variables were determined to be statistically significant. Multivariate analysis identified that lactate [Odds ratio (OR) = 1.787, 95% CI, 1.192–2.785], Tmax (OR = 11.290, 95% CI, 4.369–32.129), and CPB time (OR = 1.015, 95% CI, 1.004–1.026) were independent predictors for POD (Table 2).

**Table 2:** Multivariable logistic regression analysis of independent risk factors for POD after cardiac surgery.

Variables	β	OR	95% CI
Lactate	0.581	1.787	1.192–2.785
Tmax	2.423	11.290	4.369–32.129
CPB (min)	0.014	1.015	1.004–1.026

β: regression coefficient; OR: odds ratio; 95% CI: 95% confidence interval; CPB: cardiopulmonary bypass.

### 3.3. Model performance and internal validation

**Figure 2:**
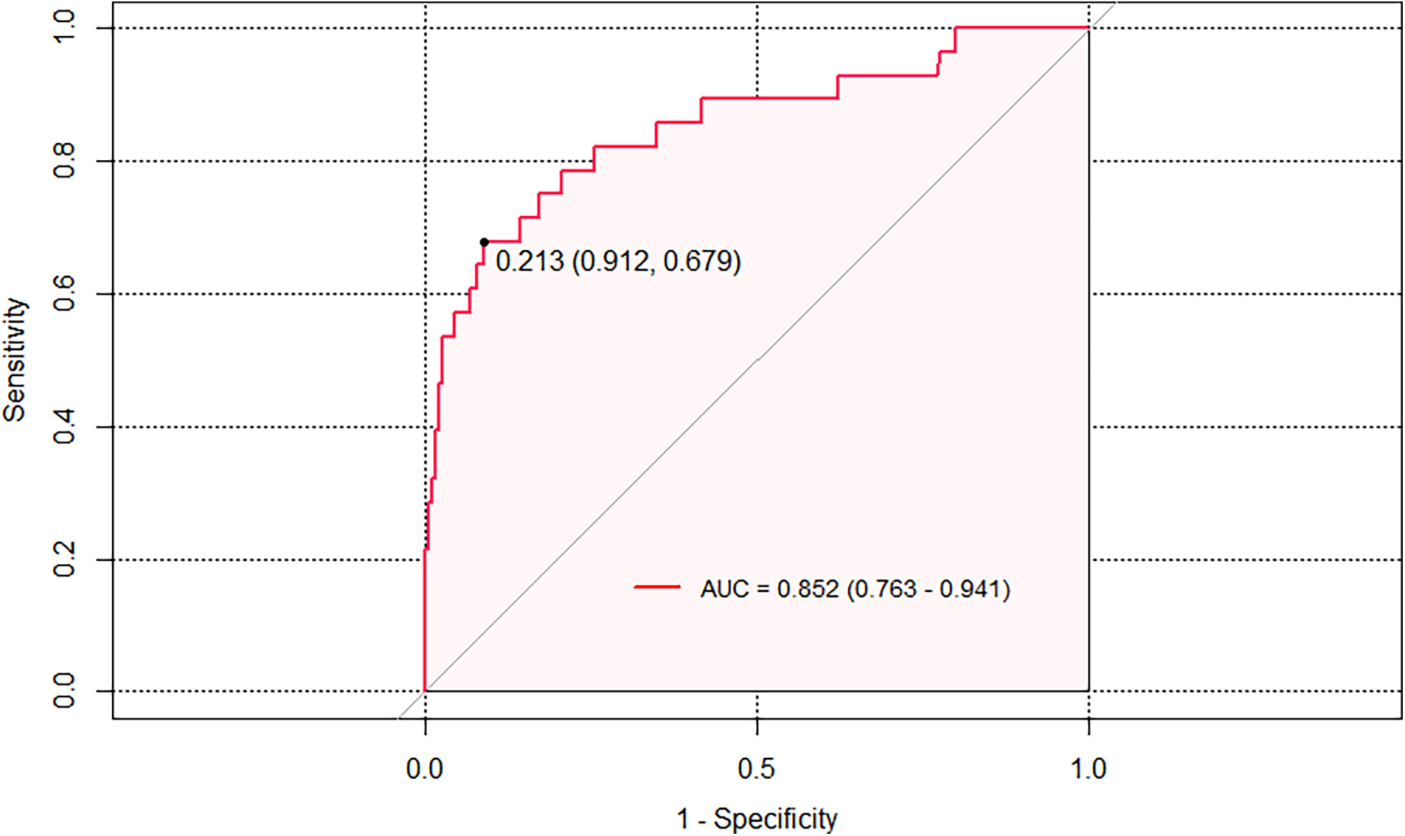
AUROC curve analysis of the model for POD; AUROC = 0.852 which is equal to a c-statistic.

**Figure 3:**
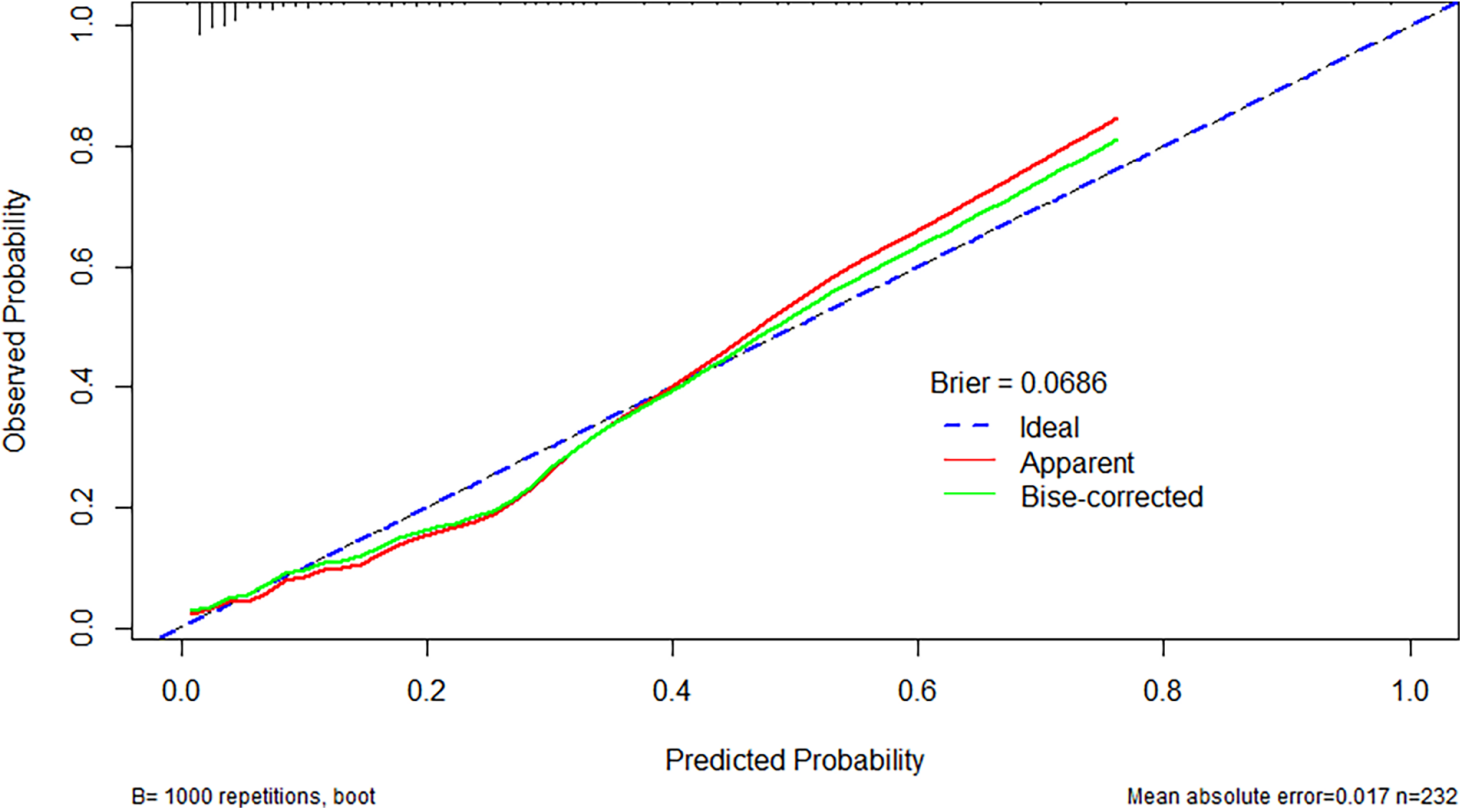
The brier score of the model was 0.0686, indicating good calibration.

**Figure 4:**
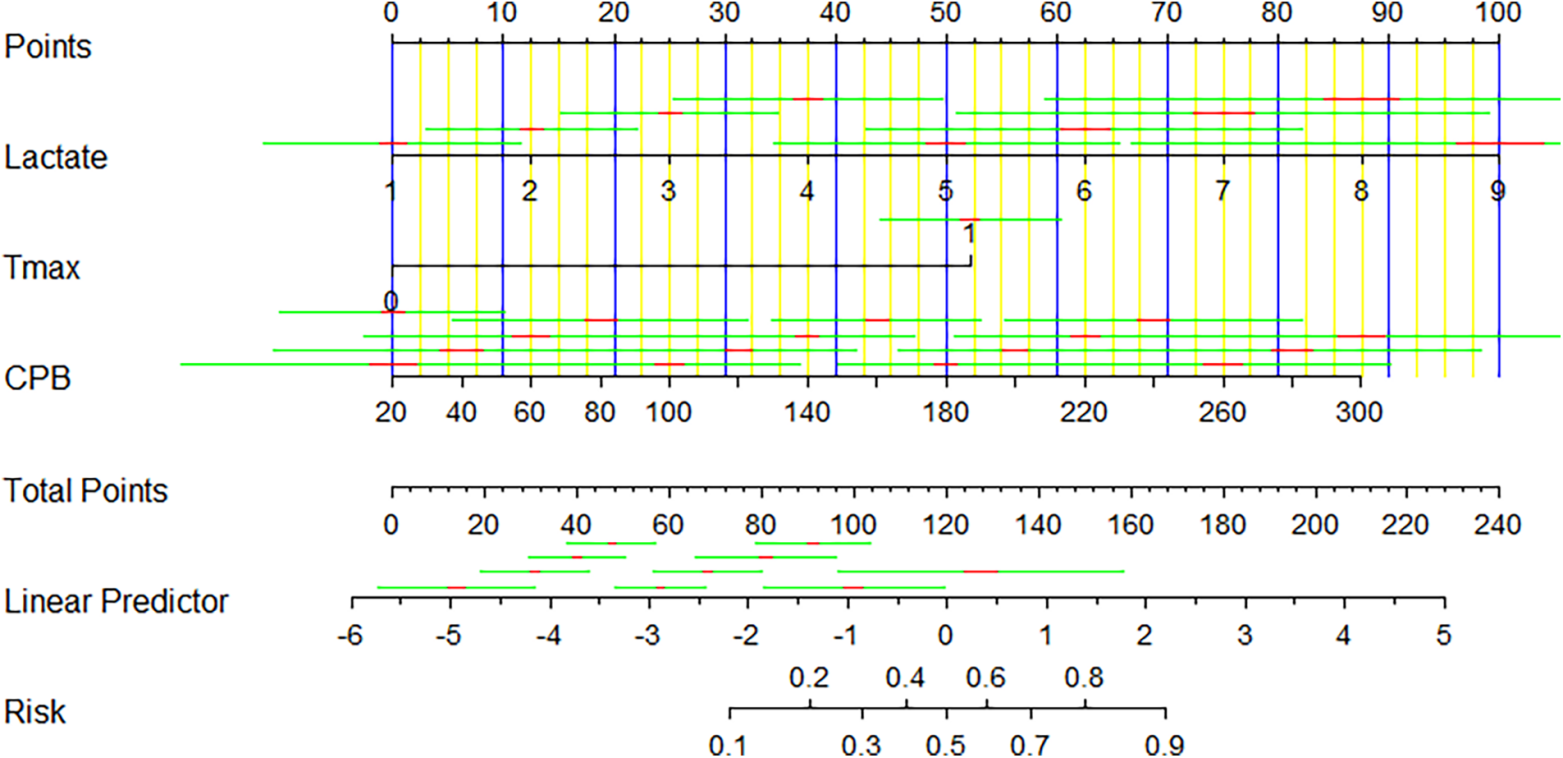
Diagnostic nomogram of model for predicting POD after cardiac surgery.

**Figure 5:**
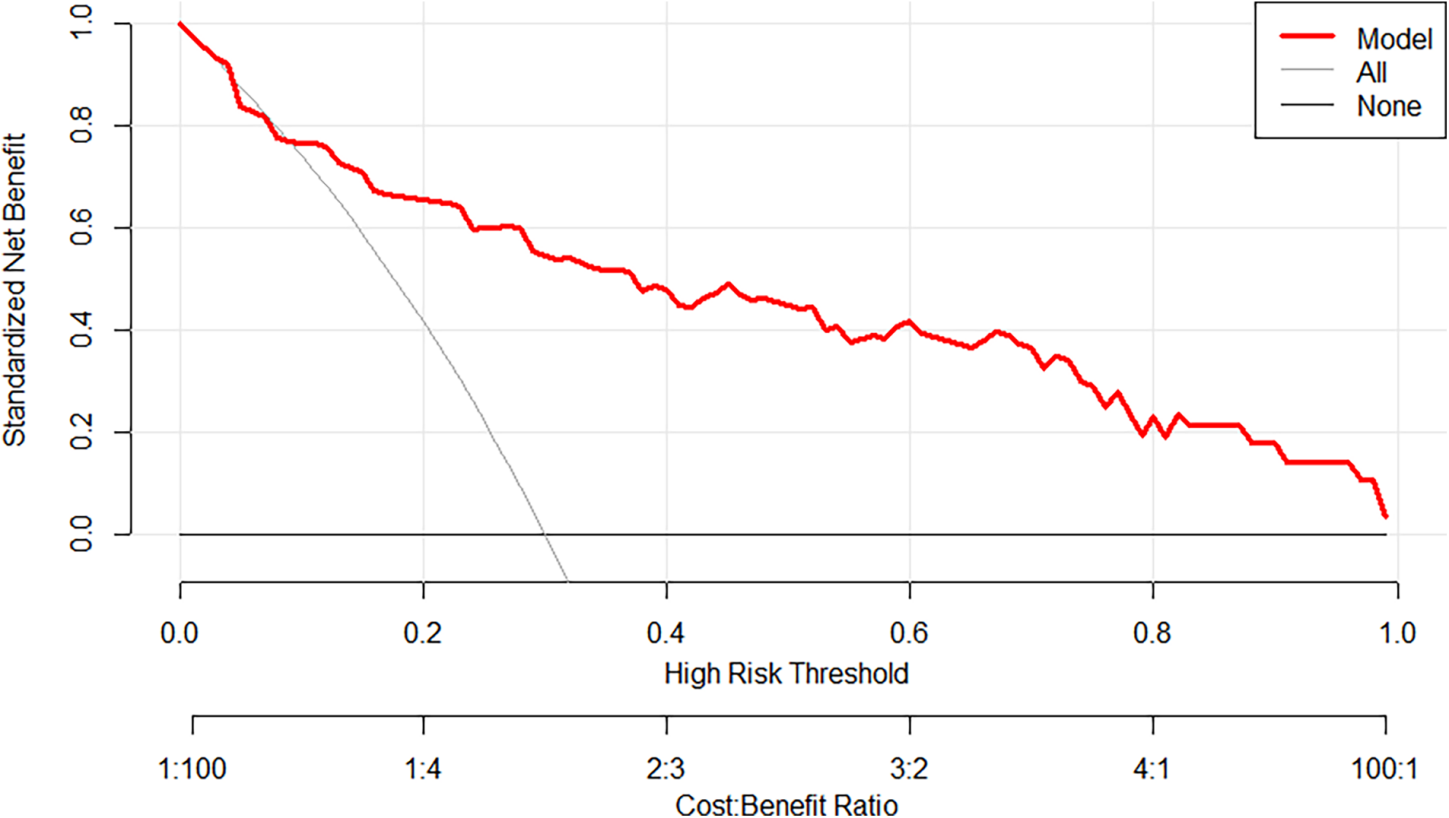
Decision curve analysis (DCA) for the study's nomogram.

The prediction model exhibited a C-statistic value of 0.852 (95% CI, 0.763–0.941), demonstrating excellent discriminatory power. Sensitivity and specificity, based on the area under the receiver operating characteristic (AUROC) curve, were 91.2% and 67.9%, respectively (Figure 2). The apparent calibration curve closely approximated the ideal 45° line, indicating consistent agreement between observed and predicted probabilities within the development cohort (Figure 3). To mitigate any potential overoptimism in the model, internal validation using the 1,000 bootstrap approach was performed, which confirmed its robust discrimination ability with a Brier score of 0.0686 (Figure 3). Utilizing these three candidate variables, we constructed a nomogram for predicting the probability of postoperative delirium (Figure 4). Furthermore, decision curve analysis demonstrated a favorable net clinical benefit (Figure 5).

## 4. Discussion

Postoperative fever after cardiac surgery is a well-known phenomenon (22, 23). It primarily arises from surgical tissue injury and the release of pro-inflammatory cytokines during CPB (22, 24, 25). Consequently, the etiology of fever in the majority of cases is non-infectious (26), particularly within the first 48 h post-surgery (27). While infections appear to be relatively rare (22, 23, 28), they represent a significant complication for patients with implanted cardiac prosthetic devices, being associated with high incidence and mortality rates (27, 29). Therefore, empirical antibiotic therapy is frequently initiated in patients experiencing postoperative fever after cardiac surgery, especially when prosthetic materials are used, potentially leading to overtreatment. Despite the recognized significance of CPB in the development of postoperative inflammation and fever following certain types of cardiac surgeries involving prosthetic materials, a comprehensive evaluation of the natural course of postoperative inflammation and fever related to CPB and implanted prosthetic materials is yet to be undertaken.

Although postoperative fever following cardiac surgery is a common occurrence (30), with an incidence as high as 38%, its etiology and significance remain incompletely understood (31). There are competing theories regarding its underlying pathophysiology. Fever may reflect an inflammatory response, which could be attributed to the surgical trauma itself or to the interaction between blood and foreign surfaces within the cardiopulmonary bypass (CPB) circuit (25, 32). Additionally, fever may serve as an indicator of altered hypothalamic thermoregulatory center function, signaling brain injury (33, 34).

Regardless of the underlying causes, fever has been demonstrated to be associated with adverse postoperative complications (25), including unfavorable cerebral outcomes (34, 35). In a recent study involving 300 patients, we described a significant relationship between postoperative hyperthermia at 6 weeks following coronary artery bypass graft surgery and decline in neurocognitive function (25). The association between fever and stroke following bypass surgery remains unclear. However, even mild hyperthermia (1–2°C) in non-cardiac surgical cases has been shown to result in poorer outcomes, such as increased infarct size, deteriorating neurological function, and elevated mortality rates (25, 36).

The fluctuation in serum lactate levels serves as a reliable prognostic biomarker for mortality in critically injured patients (37). Another study demonstrates that lactate values reflect ischemia reperfusion more rapidly and reliably than novel biomarkers (38). Elevated lactate levels in cardiac surgery patients during the perioperative period are associated with adverse postoperative outcomes. Perioperative lactate levels can serve as a predictor for the occurrence of POD in elderly trauma patients (39, 40). In our study, postoperative hyperlactatemia reflects the circulatory state during surgery and therefore correlates with the development of POD. Our research findings are consistent with these study results.

## 5. Conclusion

In conclusion, our study findings indicate that POD is highly prevalent among cardiac surgery patients. Postoperative lactate levels, cardiopulmonary bypass duration, and postoperative fever emerge as independent predictive factors for the development of postoperative delirium. Furthermore, the identification of postoperative fever, which is one of the intraoperative variables, may be related with the occurrence of POD.

## 6. Limitation

In summary, our study has some limitations that should be taken into consideration when interpreting the findings. Firstly, our study was performed at a single center, which could potentially restrict the generalizability of the findings. Secondly, the relatively small sample size could have an impact on the statistical power of the study. Additionally, the study's observational design prevents the establishment of causal relationships, and there may be additional factors not considered in the analysis. External validation of the predictive nomogram is necessary before its clinical implementation.

## Data Availability

The original contributions presented in the study are included in the article/**Supplementary Material**, further inquiries can be directed to the corresponding authors.

## References

[1] Naik R Ely E Elavarasi A. Sedoanalgesia and delirium. In: Text book of critical care. New Delhi: Jaypee (2016). p. 116–30.

[2] Evans AS Weiner MM Arora RC Chung I Deshpande R Varghese R Current approach to diagnosis and treatment of delirium after cardiac surgery. Ann Card Anaesth. (2016) 19:328–37. 10.4103/0971-9784.179634 27052077 PMC4900348

[3] Santos FS Velasco IT Fráguas R Jr. Risk factors for delirium in the elderly after coronary artery bypass graft surgery. Int Psychogeriatr. (2004) 16:175–93. 10.1017/S1041610204000365 15318763

[4] Hudetz JA Patterson KM Iqbal Z Gandhi SD Byrne AJ Hudetz AG Ketamine attenuates delirium after cardiac surgery with cardiopulmonary bypass. J Cardiothorac Vasc Anesth. (2009) 23:651–7. 10.1053/j.jvca.2008.12.021 19231245

[5] Smith LW Dimsdale JE. Postcardiotomy delirium: conclusions after 25 years? Am J Psychiatry. (1989) 146:452–8. 10.1176/ajp.146.4.452 2929744

[6] Prakanrattana U Prapaitrakool S. Efficacy of risperidone for prevention of postoperative delirium in cardiac surgery. Anaesth Intensive Care. (2007) 35:714–9. 10.1177/0310057X0703500509 17933157

[7] Afonso A Scurlock C Reich D Raikhelkar J Hossain S Bodian C Predictive model for postoperative delirium in cardiac surgical patients. Semin Cardiothorac Vasc Anesth. (2010) 14:212–7. 10.1177/1089253210374650 20647262

[8] Murray AM Levkoff SE Wetle TT Beckett L Cleary PD Schor JD Acute delirium and functional decline in the hospitalized elderly patient. J Gerontol. (1993) 48:M181–6. 10.1093/geronj/48.5.m181 8366260

[9] McCusker J Cole M Dendukuri N Belzile É Primeau F. Delirium in older medical inpatients and subsequent cognitive and functional status: a prospective study. CMAJ. (2001) 165:575–83. 11563209 PMC81415

[10] Eden BM Foreman MD. Problems associated with underrecognition of delirium in critical care: a case study. Heart Lung. (1996) 25:388–400. 10.1016/S0147-9563(96)80082-3 8886815

[11] Morency CR Levkoff SE Dick KL. Thorofare, NJ: SLACK Incorporated (1994). Vol. 20. p. 24–30. 10.3928/0098-9134-19940801-06 8077626

[12] Tan MC Felde A Kuskowski M Ward H Kelly RF Adabag AS Incidence and predictors of post-cardiotomy delirium. Am J Geriatr Psychiatry. (2008) 16:575–83. 10.1097/JGP.0b013e318172b418 18591577

[13] Koster S Oosterveld FG Hensens AG Wijma A van der Palen J. Delirium after cardiac surgery and predictive validity of a risk checklist. Ann Thorac Surg. (2008) 86:1883–7. 10.1016/j.athoracsur.2008.08.020 19022003

[14] Keijmel SP Zwartkruis IM Jongenotter J Geuzebroek GS Kouijzer IJ. Postoperative inflammation and fever after elective aortic valve and aortic root replacement: a retrospective cohort study. Open Forum Infect Dis. (2023) 10:ofad015. 10.1093/ofid/ofad015 36726552 PMC9887259

[15] Gao Y Gong S Zhou W Li X Gan X. Frequency and risk factors of subsyndromal delirium in the intensive care units: a prospective cohort study. Neuropsychiatr Dis Treat. (2023) 19:1003–16. 10.2147/NDT.S407156 37144142 PMC10153435

[16] Brown CH IV. Delirium in the cardiac surgical intensive care unit. Curr Opin Anaesthesiol. (2014) 27:117. 10.1097/ACO.0000000000000061 24514034 PMC4156112

[17] Inouye SK van Dyck CH Alessi CA Balkin S Siegal AP Horwitz RI. Clarifying confusion: the confusion assessment method. A new method for detection of delirium. Ann Intern Med. (1990) 113:941–8. 10.7326/0003-4819-113-12-941 2240918

[18] Ely EW Margolin R Francis J May L Truman B Dittus R Evaluation of delirium in critically ill patients: validation of the confusion assessment method for the intensive care unit (CAM-ICU). Crit Care Med. (2001) 29:1370–9. 10.1097/00003246-200107000-00012 11445689

[19] Pang Y Li Y Zhang Y Wang H Lang J Han L Effects of inflammation and oxidative stress on postoperative delirium in cardiac surgery. Front Cardiovasc Med. (2022) 9:1049600. 10.3389/fcvm.2022.1049600 36505383 PMC9731159

[20] Pabinger I van Es N Heinze G Posch F Riedl J Reitter EM A clinical prediction model for cancer-associated venous thromboembolism: a development and validation study in two independent prospective cohorts. Lancet Haematol. (2018) 5:e289–98. 10.1016/S2352-3026(18)30063-2 29885940 PMC7338218

[21] Steyerberg EW Vergouwe Y. Towards better clinical prediction models: seven steps for development and an ABCD for validation. Eur Heart J. (2014) 35:1925–31. 10.1093/eurheartj/ehu207 24898551 PMC4155437

[22] Livelli FD Jr. Johnson RA McEnany MT Sherman E Newell J Block PC Unexplained in-hospital fever following cardiac surgery. Natural history, relationship to postpericardiotomy syndrome, and a prospective study of therapy with indomethacin versus placebo. Circulation. (1978) 57:968–75. 10.1161/01.cir.57.5.968 346257

[23] Andrade C Olvera S Reyes P. Fever and infection after heart surgery. A prospective study of 75 cases. Arch Inst Cardiol Mex. (1989) 59:487–91. 2604490

[24] Warltier DC Laffey JG Boylan JF Cheng DC. The systemic inflammatory response to cardiac surgery: implications for the anesthesiologist. J Am Soc Anesthesiol. (2002) 97:215–52. 10.1097/00000542-200207000-00030 12131125

[25] Mitchell JD Grocott HP Phillips-Bute B Mathew JP Newman MF Bar-Yosef S. Cytokine secretion after cardiac surgery and its relationship to postoperative fever. Cytokine. (2007) 38:37–42. 10.1016/j.cyto.2007.04.009 17572096

[26] Mookhoek A Korteland NM Arabkhani B Di Centa I Lansac E Bekkers JA Bentall procedure: a systematic review and meta-analysis. Ann Thorac Surg. (2016) 101:1684–9. 10.1016/j.athoracsur.2015.10.090 26857635

[27] Rhee C Sax PE. Evaluation of fever and infections in cardiac surgery patients. Semin Cardiothorac Vasc Anesth. (2015) 19:143–53. 10.1177/1089253214538524 24958717

[28] Miholic J Hiertz H Hudec M Laczkovics A Domanig E. Fever, leucocytosis and infection after open heart surgery. A log-linear regression analysis of 115 cases. Thorac Cardiovasc Surg. (1984) 32:45–8. 10.1055/s-2007-1023343 6198774

[29] Machelart I Greib C Wirth G Camou F Issa N Viallard JF Graft infection after a Bentall procedure: a case series and systematic review of the literature. Diagn Microbiol Infect Dis. (2017) 88:158–62. 10.1016/j.diagmicrobio.2017.03.002 28330738

[30] O’Mara SK. Management of postoperative fever in adult cardiac surgical patients. Dimens Crit Care Nurs. (2017) 36:182–92. 10.1097/DCC.0000000000000248 28375995

[31] Thong WY Strickler AG Li S Stewart EE Collier CL Vaughn WK Hyperthermia in the forty-eight hours after cardiopulmonary bypass. Anesth Analg. (2002) 95:1489–95. 10.1097/00000539-200212000-00006 12456406

[32] Hindman BJ. Emboli, inflammation, and CNS impairment: an overview. Heart Surg Forum. (2002) 5:249–53. 12538140

[33] Beamer NB Coull BM Clark WM Hazel JS Silberger JR. Interleukin-6 and interleukin-1 receptor antagonist in acute stroke. Ann Neurol. (1995) 37:800–5. 10.1002/ana.410370614 7778854

[34] Grocott HP Mackensen GB Grigore AM Mathew J Reves J Phillips-Bute B Postoperative hyperthermia is associated with cognitive dysfunction after coronary artery bypass graft surgery. Stroke. (2002) 33:537–41. 10.1161/hs0202.102600 11823666

[35] Cook DJ. Cerebral hyperthermia and cardiac surgery: consequences and prevention. Semin Thorac Cardiovasc Surg. (2001) 13:176–83. 10.1053/stcs.2001.23557 11494209

[36] Reith J Jorgensen HS Pedersen PM Nakayama H Raaschou HO Jeppesen LL Body temperature in acute stroke: relation to stroke severity, infarct size, mortality, and outcome. Lancet. (1996) 347:422–5. 10.1016/s0140-6736(96)90008-2 8618482

[37] Régnier M-A Raux M Le Manach Y Asencio Y Gaillard J Devilliers C Prognostic significance of blood lactate and lactate clearance in trauma patients. J Am Soc Anesthesiologists. (2012) 117:1276–88. 10.1097/ALN.0b013e318273349d 23168430

[38] Balcı E Demir ZA Yiğit Özay H Vardar K Karduz G Aksu U Effects of upper limb ischemia-reperfusion on regional oxidative stress during aortic surgery with moderate hypothermia. J Card Surg. (2021) 36:1361–9. 10.1111/jocs.1540 33567138

[39] Ozay HY Bindal M Turkkan S Beyoglu MA Yekeler E Turan S. Delirium development after lung transplantation: an intraoperative assessment. Transplant Proc. (2022) 7:1906–12. 10.1016/j.transproceed.2022.03.069 35985880

[40] Lee C Lee J Cho H Song J Jung H Ma X The association of perioperative serum lactate levels with postoperative delirium in elderly trauma patients. BioMed Res Int. (2019) 2019:3963780. 10.1155/2019/3963780 31828102 PMC6881750

