# Supplementary figures and images for: Unveiling the nexus of postoperative fever and delirium in cardiac surgery: identifying predictors for enhanced patient care
Source: Front Cardiovasc Med. 2023 Nov 10;10:1237055. doi: 10.3389/fcvm.2023.1237055 (PMC10667695; doi:10.3389/fcvm.2023.1237055)

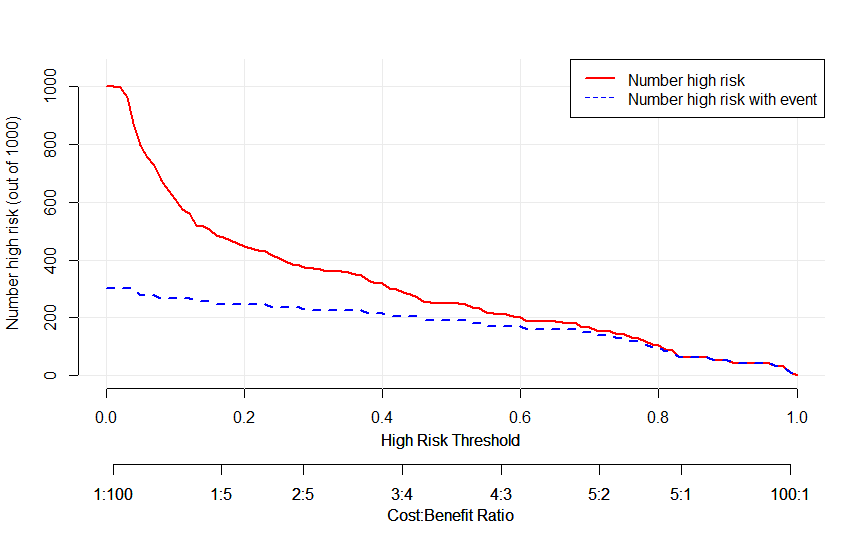

Supplement: Supplementary file 1 [file Image1.tiff]
